# An observational study on sport-induced modulation of negative attitude towards disability

**DOI:** 10.1371/journal.pone.0187043

**Published:** 2017-11-15

**Authors:** Giovanni Ottoboni, Melissa Milani, Annalisa Setti, Andrea Ceciliani, Rabih Chattat, Alessia Tessari

**Affiliations:** 1 Department of Psychology, University of Bologna, Bologna, Italy; 2 Department for Life Quality Studies, University of Bologna, Bologna, Italy; 3 School of Applied Psychology, University of Cork, Cork, Ireland; Universita degli Studi di Perugia, ITALY

## Abstract

The present study investigates whether sport activities involving children with a disability can reduce negative attitudes towards disability in children without disability. We compared the effect of being schoolmate or member of the same football team whereby a child with disability was member of the class/team or not. This lead to four groups that were assessed both at the beginning and at the end of the school year. Two measures were collected: an ad-hoc questionnaire and an Implicit Association Test. The two assessments were designed to measure explicit and implicit attitudes towards children with disabilities. Results suggested that sport activities over one school year reduced more (p < .001) the implicit negative attitude towards disability (Mean = -.17, sd = .10) than the contact with the classmate in the school context (Mean = -.03, sd = .14), possibly due to their team building characteristic.

## Introduction

Disability is a multidimensional experience and, by virtue of its complexity, it is a heavily debated issue [1]. While current theoretical and cultural models, i.e., the medical and the psychosocial, focus on the reasons beyond and the solutions to contrast difficulties and discriminations encountered by people with disability, it is fundamental to keep the person at the core of the debate [2]. Indeed, persons with disabilities deserve respect for their functional limitations, as they require specific functional adaptation, e.g. restrictions and exertions, and to balance their special health conditions with contextual environmental and personal factors [3]. This constant adaptation process, and the challenges related to it, must be taken into account so that individuals with disability can be finally accepted as persons who, while having disabilities, are active part of the society [1].

The education system has a primary role in our society in driving people’s opinion towards the personal aspects of disability [4]. School endorses (or at least it should endorse) its primary role of fostering many virtues, among which, one of the most important regards the respect of others. To achieve such an ambitious goal, the educational system should encourage children to acquire and develop knowledge about persons with disabilities. Knowledge is indeed one of the most powerful tools society can adopt to minimize the negative attitudes towards those who are considered out-members, and this is the case for people with disabilities [5, 6].

The objective of increasing the *knowledge of others* may be fulfilled by adopting different strategies; one of them concerns with gathering together members belonging to different groups so that they could experience real contact with the members of “*other*” group [7]. Pettigrew and Tropp [8] reported that the positive effect of intergroup contact, while being the most commonly investigated strategy, is not always effective. At the end of their study, the authors reported that *knowledge of others*, *intergroup contact* and *empathy* modulate the prejudice against the others. The authors indicated that the simplistic knowledge about characteristics of persons with disability did not produce positive outcomes, as it does, instead, the specific action aimed at reducing anxiety in in-group members. The reduction of anxiety is particularly important in the initial moments of contact. The authors concluded that actions addressing empathy and perspective taking have positive impact against prejudice: such actions produce the widest effect, followed by actions focused on reducing anxiety and then by mere contact. The fundamental role of mediators to inter-group contact was also evident in Krahé and Altwasser [9]. Working to decrease the prejudices against physical disabilities, there authors noted that by offering adolescents’ the chance to engage in collaborative sport activities with athletes with disability (i.e., as playing in the same team), the attitudes towards people with disabilities tended to decrease more than in another group of participants who had a group conversation about disabilities. Even though the dimension of personal contact was not taken into account in the control group, what emerged in the study is that the collaborative nature of the sport activity may have played a very important role. Indeed, when people are placed in cooperative or interdependent settings, this positively affects their attitudes towards persons with disability. However, despite the manifold evidence, the assumption that contact redefines out-group-directed bias is still under debate because the evidence in support are not definitive [10, 11]. The several reasons causing such dimorphism may have to do with the differences in the cultural backgrounds accessed, the assessments carried, the interventions deployed or the cohorts investigated [12]. For what concerns the latter issue, age holds a weight in defining the level of prejudice by acting as prognostic of negative attitude [13]. *Social domain theory* assumes that the younger the children, the lesser they are negatively biased towards deviant out-group members [14]. One of the reason concerns the visual salience of the disability: Disabilities tend to affect a low percentage of people; thus, their relatively low frequency might increase the cognitive presence and phenomena’s mental accessibility of children representative in the world. Recently, however, Huckstadt and Shutts [15] reported that children aged between 3 and 5 years tend to prefer more pictures of typically developing children than pictures of children with disabilities. At the same time, when they were requested to express their preference verbally, they judged better children with disabilities than children who were typically developing but described as having committed mild crimes. Such evidence seems to suggest another cause undermining the bias: the persons with disabilities tend to be considered as having low competence [16].

In the present study, we aimed at clarifying the impact of cooperative social activities on the modulation of implicit and explicit prejudice towards peers with disabilities. Specifically, we investigated the impact of one year spent with a child with disability. The aim was achieved by studying two different contexts: school activities and team sport activities. School activities were not particularly targeted to increase students’ positive attitudes towards the classmate with disability, even if we cannot exclude that some mention of inclusion issues, racial bias or disability-related issues occurred during classes. On the other hand, the team sport context, specifically football, explicitly foster cooperation, as collaboration amongst participants is fundamental to win. Lai and colleagues [17] hhighlighted that among the many improvements that contact-related exposure may cause, a very promising was the one that involved participants involved cooperation with persons with disability. However, despite the described beneficial effects, the authors [17] were not able to control for the time consistency of the effect, as they tested (adult) participants exposed to persons with disability only for a limited time. As the effect of interventions tend to decay over time, we tested a longer period of exposure with a pre-adolescent cohort.

We tested four groups of participants, each of them was characterized either by the presence or the absence of contact with a school/team mate with disability. All the groups were assessed by measuring the implicit level of prejudice by the Implicit Association Test (IAT), followed by a brief, ad-hoc developed, questionnaire to measure explicit prejudice. Very recently, indeed, contact has been suggested to reduce implicit attitudes described from the output of an IAT test (See *The IAT* paragraph, for relative references).

The concept of implicit attitude is well-described by Greenwald, McGhee and Schwartz [18] when they indicate that “*the difference between implicit and explicit attitudes manifested itself as a function of individual’s full awareness of the causes guiding the behaviour*” and it is supported by *Environmental Association Model* [19]. The model suggests that attitudes are driven by the environment in which persons live more than reflecting the association they explicitly endorse. In sum, we expect that exposure to children with disability will increase positive views and decrease prejudice on disability when involved in activities with the children with disability, and more so for collaborative activities such as sport.

## Methods

### Ethical clearance

Participants were invited to participate in the study by their teachers and football coaches. Both teachers and coaches were provided with with information on the study and with information sheets for parents. Children and parents were informed of their right to withdraw from the study at any time without penalty. Parents who requested further explanation and information were met on an individual basis. Parents signed informed consent in agreement with Declaration of Helsinki. Moreover, children were explicitly asked to verbally consent to take part into the study before commencing and reminded to the right of withdrawing. The study was approved by the UNIBO Ethics Committee, and presented “no more than minimal risk”.

### Participants

Altogether, 161 Italian children from Emilia Romagna region participated in the present study. Analyses utilized data only from 116 of them as the rest did not take part in the second session or were excluded as they did not respect the inclusion criteria indicated by Greenwald [20]. The remaining participants were aged between 10 and 12 years (Mean 11.41, sd = .60) and they were recruited from local football clubs and primary schools. Children were divided into groups which were intact groups, i.e., the composition of the groups were not controlled or composed via randomization manipulation and they overlapped with classrooms or football teams. The two football teams were both composed of beginners players. One group (Sport-With team-mate with disability: Sp-Wi) of 25 boys was composed of football players having a team-mate with disabilities (*ICD-10*: *G81.1* Spastic hemiplegia of the left side of the body, respectively + G40.2, Epilepsy and recurrent seizures, localization-related (focal) (partial) symptomatic epilepsy and epileptic syndromes with complex partial seizures, not intractable). Another group of boys (Sport-Without team-mate with disability:Sp-Wo) was composed of 33 football players who hadn’t a team-mate with disability on the team. The third group (School-With classmate with disability: Sc-Wi) was composed of 26 children (17 girls and 9 boys) belonging to the same class. They had a peer with disabilities in ther class (*ICD-10*: *F82*, Specific developmental disorder of motor function + *F81.9*, Developmental disorder of scholastic skills, unspecified + *F93*, Emotional disorders with onset specific to childhood). The fourth group (School-Without classmate with disability: Sc-Wo) was composed of 32 children, 12 girls and 20 boys, neither having a class nor sport team-mate with disabilities.

### Procedure

Experimenters met the children twice: at the beginning of the school/sport year (October, 2013—T1) and at the end of the same year (May, 2014—T2). During each meeting, children were tested with an ad-hoc designed version of IAT [21] and an ad-hoc built paper-and-pencil survey aimed at assessing explicit attitude towards peers with disability. Participants were tested in groups of 5. Each of them sat in front of a laptop computer that was placed at a distance of 40/50 cm. The survey always followed the IAT.

### Dependent measures

#### The IAT

The IAT is a computerized task capable of measuring how strong is the association between two conceptual categories (Children with disabilities, Children without disabilities) and two evaluative attributes (i.e., Good, Bad) [21].

Participants were tested following the procedures recommended by Greenwald and colleagues [22–24]. IAT was delivered by laptop computers with E-prime software [25].

**Fig 1:**
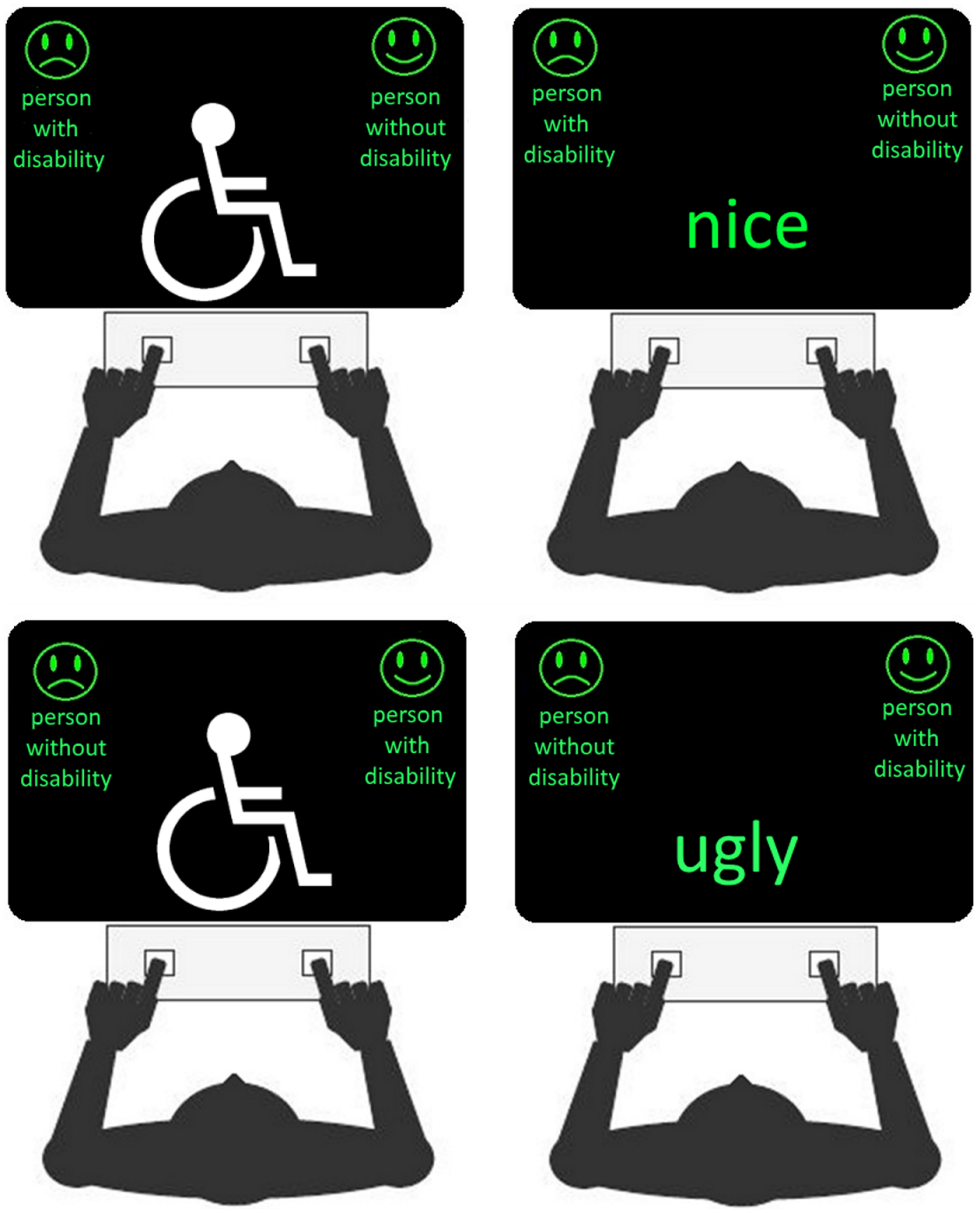
The figure depicts four examples of screens used during the critical IAT blocks. The two left panels depict examples of words stimuli; in the two panels on the right the pictures of children with disability are substituted with an icon for illustrative purpose: during the tests, instead of the icon, real images of children with or without disabilities were presented to the participants. The panels on the top row report the case of Block 3 and Block 4 where the stimulus-response association was learned by mapping the left key, pictures of children without disability and negative-valued words. The panels on the bottom row report report the case of Block 6 and Block 7 where the stimulus-response association emerged by mapping the left-key with pictures of children with disability and positive-valued words. The figures are original and not copyrighted.

Participants were instructed to be as fast and accurate as possible to provide the responses according to the instructions they received at the beginning of each block by pressing the keyboard’s keys marked with a white patch. The patches were set over “z” and “/” keys, that were the most lateral keys of the keyboards that allow a comfortable response posture. According to the different blocks, participants were instructed to press the key on their left or the one on their right at the appearance an image or a word. Block 1 (16 trials) and Block 2 (16 trials) were practice blocks: in Block 1, participants mapped each image’s category with a specific keyboard’s key (e.g., left key for children without disabilities, right key for children with disabilities); in Block 2, they did the same mapping with each evaluative attributes (e.g., left key for bad words and right key for good words; See Fig 1). The following blocks, Block 3 and 4 (both of 32 trials), served to pair categories and evaluative attributes. In these blocks, images and words appeared one after the other in random order. Participants were requested to respond to them according to the mappings they learned in Block 1- for what concerned the images- and Block 2- for what concerned the words. For instance, they pressed the left key at the view of images of children without disabilities, or at the reading of negative valued words, whereas they press the right key at the view of images of children with disabilities and positive valued words. Between Block 3 and 4 there was a break of few minutes. Block 5 (16 trials) was another practice block: it was used to re-map one of the previous associations, the one between images stimuli and response keys. In keeping with the example depicted in Fig 1, in Block 5, participants mapped the left key for children with disabilities, and the right one for children without disabilities. Block 6 and 7 (32 trials in total), participants were tested with the just learned image/key association and with the one learned previously between valued words and response key. As it happens in Block 3 and 4, the images and the words were presented in random order. However, now they mapped left key for positive words and right key for negative words. As previously, between Block 6 and 7 there was a break of few minutes. Only Block, 3, 4, 6, 7 were used to make the resulting IAT scoring. The position of the Good/Bad categories was randomized between participants: half of them mapped Good to the right key and Bad to the left one, half of them mapped the reverse. We counterbalanced also the mapping between responses and type of images, thus we ended up with 4 versions of the same script. Moreover, in order to counterbalance context-related in-group bias [26] we further generated four scripts. The former four differed from the latter by the type of images that were displayed: the participants who were presented with sport-related images in T1, were presented with images depicting standard activities in T2 and vice-versa. Within the group, the children were randomly exposed to one of the context in T1 (sport-related and standard activities), and counterbalanced in T2. The words children were asked to evaluate to have good or bad attributes were cool, smart, happy, good, boring, ugly, mean, sad [27]. The images represented children with or without disabilities involved in running, practising tennis, football or basketball. The standard activities involved children blowing bubbles, seating at the computer desk, playing with a ball in the garden, responding to some calls inside a school.

#### Scoring procedure

The IAT associative strength was calculated using a *d* algorithm as suggested by Greenward and colleagues [20]. Responses that were slower than 10,000 msec and participants who responded faster than 300 msec in the 10% of the trials were removed from the analysis. Participants were also excluded if the error rate in any critical blocks was above the 40%. Twenty-two participants were excluded because of such criteria. d-score was then calculated by averaging the difference in response latency between the critical blocks divided by the standard deviation of participants’ response latency in the same blocks (*inclusive SD*) [20]. Such calculation was performed once 600 msec were added to any responding errors participant incurred in. Moreover, due to the counterbalancing procedure we adopted, any block of trials that associate positive-valued words with images of children without disabilities was labelled as *compatible*, while as *incompatible* was labelled any the block of trials that associate the negative words with children without disabilities. In this way, since compatible responses were subtracted from incompatible responses, a larger *d*-score indicates a more negative implicit attitude towards children with disabilities.

#### The explicit assessment

The explicit attitude towards peers with disability was assessed by a paper-and-pencil survey. The instructions participants received invited them to complete the survey aimed at measuring attitudes and behavior towards disabilities. Once participants were provided with paper and pencil, both instruction and each statement were read aloud by one experimenter. At the same time, children were asked to read silently the statements and provided with the time to respond to them privately. The collective reading made the experimenter able to respond to the questions children addressed about terminology and methodology, as, for example, that they should consider paralysis, loss of vision or hearing, or mental disabilities as disabilities. Participants were asked to indicate the level of agreement they felt with each statement. The agreement scale was a 5 points scale that went from “Strongly agree” to “Strongly disagree”. The ratings expressed for each statement were then scored from 4 to 0 and averaged across the statements in order to obtain an index with the higher values to indicate the positive attitudes. The score for statements number 5, 6, 7, 8, 10 were reversed (S1 File).

## Statistical methods

R-software was used for all statistical analysis and the bar chart [28]. In order to evaluate whether one year spent with or without engagement with a mate, classmate or team-mate, with disabilities modulated or not either the implicit and the explicit attitudes towards peers with disabilities, we calculated two resulting indexes. We subtracted either the implicit and the explicit scores we measured at T1 from the ones we measured in T2. For what concerns the IAT, we called the resulting index IAT Δ*d-score*; for what concerns the explicit questionnaire we called Explicit Δ*d-score*. The resulting IAT and Explicit Δ*d-score* were then entered as measured variable into different Univariate Analysis of Variance both accounting the between-subject factors *Presence* (of a peer with disabilities within the class- team) and *Setting* (Football club—School class). Tukey tests for multiple comparisons of means were used for post-hoc analysis and to provide adjusted p-values. Significance level was set to.05. The dataset supporting the article has been uploaded as part of the S2 File.

## Results

**Fig 2:**
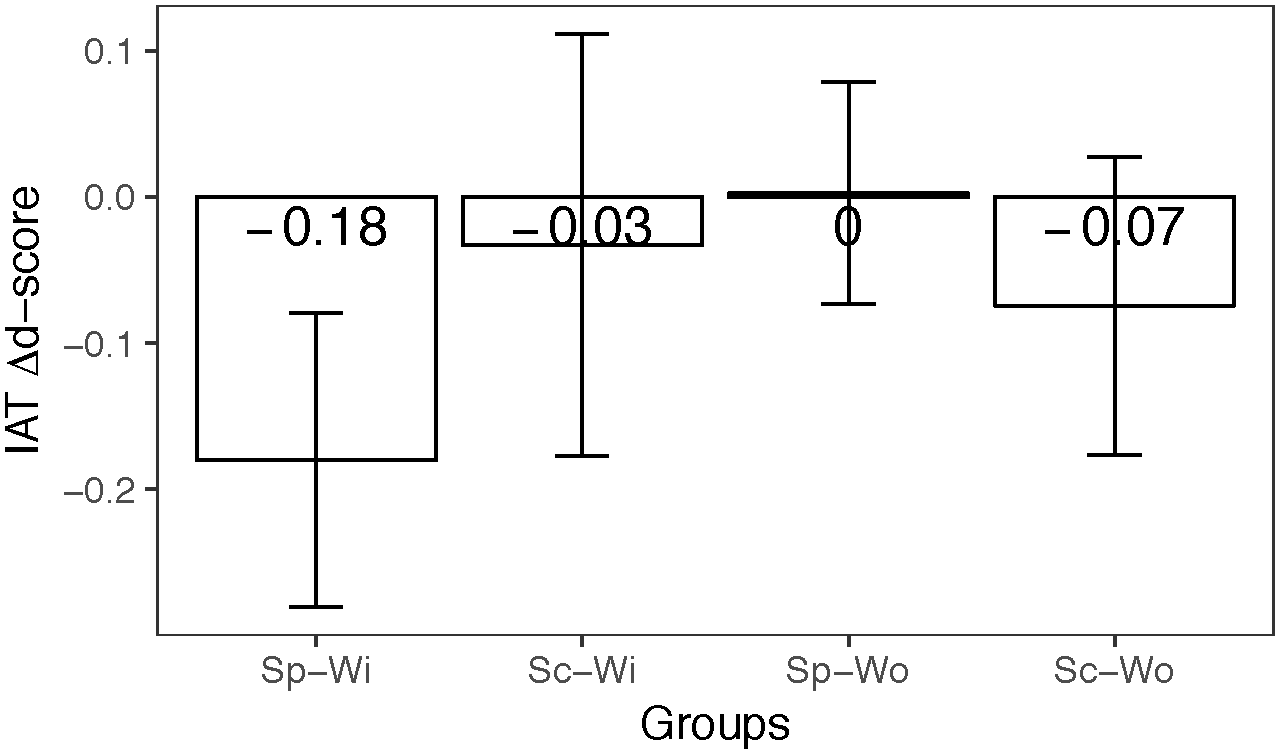
The figure represents the average IAT of each experimental group. Error bars represent the standard deviation values.

The IAT Δ*d-score* analysis revealed no difference between the *Setting* (Football setting: Mean = -.076, sd = .13; School setting: Mean = -.055, sd = .13); F(1, 112) = 1.05, $\eta_{p}^{2}=.010$, p = .31), but that the presence of a mate with disability in the team or in the class was associated with a larger decrease in the *d-score* than when this was not the case (Presence: Mean = -.10, sd = .14; Absence: Mean = -.035, sd = .10); F(1, 112) = 12.37, $\eta_{p}^{2}=.10$, p < .001). Furthermore, and more important for the purpose of the present work, the interaction between *Presence* and *Setting* was significant (F(1, 112) = 31.55, $\eta_{p}^{2}=.22$, p < .001, See Fig 2). The Tukey post-hoc analysis revealed that Sp-Wi reported a modulation in the implicit attitude wider than the other groups. Indeed, the comparison between Sc-Wo and Sp-Wo (p = .02), between Sp-Wi and Sp-Wo (p < .001), between Sp-Wi and Sc-Wo (p = .002) and between Sc-Wi and Sp-Wi (p < .001) were all significant, while they were not the differences between Sc-Wi and Sp-Wo (p = .6) and between Sc-Wi and Sc-Wo (p = .46). The Explicit Δ*d-score* analysis revealed that neither main effects or interaction were significant (Ps > .05; See S3 File for further analysis).

## Discussion

The results supported the hypothesis that sport has a role in reducing the implicit negative attitude towards the out-groups, specifically towards children with disabilities when the sport activities are played together with a team-mate having some form of disabilities. Regarding the explicit judgements, instead, the results did not show the same positive change. The sport we tested was a team sport where the collaboration among the team-mates is fundamental to achieve the goal of victory. In the sport domain, the team-building policy passes trough the formation of boundaries capable to differentiate the in-group from the out-group. As well described by Abrams Killen), social exclusion is encouraged when it is functional to the achievements of the group’s goals. In team sport, for example, the social exclusion is legitimated by the nature of the sport itself, even though it is not based on prejudice or membership preference. In the present study, the fact that the child with disability had an active role in the team by playing as a goal-keeper reserve plausibly had multiple positive repercussions on the team mates, which produced the positive effect we measured. Indeed, as pointed out by Hinde [29]), a history of positive interactions produces positive outcomes, independently of whether the hypothesised goals are successfully attained or not. When children with disabilities participate in sport activities, their perceived physical competence and social acceptance increase. Indeed, it has been shown that by working both on the personal and on the social side, one can obtain a positive effect on what children with disabilities think of themselves, as well as on what outgroup members think about the person with disabilities [30, 31].

The *Environmental Association Model* argues that the repeated exposure to mostly negative representations of people with disabilities is what produces negative implicit attitudes [32]. In this light, our findings indicate that the same can occur in terms of increasing positive views: the more people experience positive situations concerning qualities of people with disabilities, the more the negative bias towards them can be reduced. Differently, Cohesion theory proposes that the achievement of positive social relationships among the in-group and the out-group depends on the group’s norms. According to the level of general support that norms achieve within the peers group, in-group individuals may behave and react differently to the out-group components. In regards of, for example, school achievements, when norms attempt to promote equalitarian levels of achievement, secondary social goals might be positively achieved even without intention. In this light, the present results suggest that the contact achieved through the cooperative experiences proper of a team sport as football, can lead to positive outcomes and that, as long as contact is neither infrequent or negative, beneficial effects might rise for what concerns implicit attitudes.

Analogues reccomendations were addressed in Grenier, Collins, Wright, and Kearns [33], where the practise of sport for people with disability by using the same devices they would be used to use, i.e., practising sport as people with disability do, that is using wheelchairs to play basketball, or handling short stick to play hokey, leads young children to consider the persons with disability just like the others, i.e., able to practice sport at high level.

On the other side, *Conflict theories* suggest that school-related academic goals might work to inhibit optimal achievement [34–36]. The conflict perspective can be seen as suggesting that peers with disabilities could potentially distract other team and classmate from the achievement of their goals, and pressure them to decide between social pursuits and tasks to achieve. Even if our results cannot support the conflict perspective because of insufficient knowledge of the daily school activities in this respect, we can assume that due to the specific (e.g., team-focused) nature of the sport, social pursuit and sport aim are aligned. Furthermore, by taking into account the role that saliency plays in modulating the approach of children to disability [14] we could speculate that it played a role also in the determining our results. The visibility of the disability that children have experienced—as a function of the diagnosis their respective class and teammate received—could have influenced the modulation of the level of implicit attitudes. The explicit collaborative nature of sport may have also affected children’s bias: during sport as football, individual activities are usually fewer and less important than group activities. Therefore, in a sports context, the disability may have been experienced as potentially less salient and noticeable in comparison to the school context, where higher emphasis is put on individual performance. Moreover, by taking into account both that children tend to associate the concept of disability with concepts as unpleasantness and illness [37], and that sport and health status tend to correlate strongly [38–40] our result of a baseline higher level of negative bias in the sport context is plausible. Considering then the social nature of football, then the decrease after dealing with the child with disability can also be explained within this framework. Generally, the adoption of cooperative learning structure (i.e., when students are instructed to teach and learn from one another, as during the learning of a new motor skill) leads to higher achievements than using competition or individual strategies. Indeed, as Roseth, Johnson, and Johnson [41] suggested, the resulting positive relation between cooperative learning and achievement can come out as a by-product of the cooperative attitudes, but also it can be one of the necessary conditions to achieve high level of achievement. Disability is, in fact, a multidimensional experience. From the point of view of the out-group observers, it might be seen as a state of decreased functioning associated with disease, disorder, injury, or other health conditions [24, 37]. From the point of view of the person with disability it might is experienced as an impairment that limit activities in specific context by producing participation restrictions. By adopting this psico-social-reminding perspective, health and environmental aspects featuring disability might be used to tailor and personalize activities to improve the sense of functioning by improving the participation of people with disabilities [3].

## Conclusion

The present work was aimed at clarifying whether the cooperative nature of social activities might reduce the levels of either implicit or explicit negative attitudes towards peers with disabilities. The results indicated that practising sport with team-mate with disability does have a positive impact over the rest of the team. However, this was particularly clear only in the difference between the two IAT implicit scores. The level of explicit attitudes did not change over time. This discrepancy has been previously found in the literature [42]. The study has a number of limitations that need to be controlled for in further studies. The first concerns the fact that we were not able to control for the number of school mates with disabilities each football club children meet everyday at school. However this is true for both the two football clubs, and the group of school mates without disabilities, therefore constituting a random error. A second limitation is related to the number activities had with team/classmate with disability. The children attending the football club might have had more visceral activities (run, sweat, had showers) compared to the activities that normally are attended in school; such activity might have been so visceral to have increased the level of respective deep knowledge. Despite its limitations, the results indicate that team sports may be an effective way to combat negative stereotype and proposes a practical avenue to evaluate them.

## Supporting information

S1 File Explicit questionnaire. The questions composing the Explicit questionnaire are reported in table.
(PDF)

S2 File Dataset. The data that were analysed are reported as a function of the experimental variables for each participant.
(XLSX)

S3 File Statistical supplementary materials. The material includes supplementary analysis performed to better describe the phenomena reported in the paper. In particular, the analysis that were performed on the IAT data collected in T1 are included together with the analysis concerning the internal consistency of the explicit questionnaire, the analysis performed on the Explicit Δ*d-score*, and the one performed on the Explicit data collected in T1. The last paragraph regards the correlation analysis between implicit and explicit data.
(PDF)
