# Supplementary material for: An observational study on sport-induced modulation of negative attitude towards disability
Source: PLoS One. 2017 Nov 15;12(11):e0187043. doi: 10.1371/journal.pone.0187043 (PMC5687735; doi:10.1371/journal.pone.0187043)
Supplement: S1 File — The questions composing the Explicit questionnaire are reported in table. (PDF) [file pone.0187043.s001.pdf]

N	Statement	Strongly Agree	Agree	Uncertain	Disagree	Strongly Disagree
1	My heart goes out to people in wheelchairs	SA	A	U	D	SD
2	I feel sympathetic toward people who are visually disabled	SA	A	U	D	SD
3	I assume that people with disabilities deserve special consideration	SA	A	U	D	SD
4	I am more understanding of physical or sensory disabilities than mental ones	SA	A	U	D	SD
5	People who look or act differently scare me	SA	A	U	D	SD
6	I sometimes think that people who claim to have emotional problems are faking it	SA	A	U	D	SD
7	I sometimes feel that people with disabilities have been punished by God for something they did	SA	A	U	D	SD
8	I tend to talk with people with disabilities in a different tone of voice	SA	A	U	D	SD
9	I tend to be more patient with people with disabilities	SA	A	U	D	SD
10	I get angry more quickly at people with disabilities	SA	A	U	D	SD

Table 1: The table reports the questions of the Explicit questionnaire.
