# Supplementary material for: An observational study on sport-induced modulation of negative attitude towards disability
Source: PLoS One. 2017 Nov 15;12(11):e0187043. doi: 10.1371/journal.pone.0187043 (PMC5687735; doi:10.1371/journal.pone.0187043)
Supplement: S3 File — The material includes supplementary analysis performed to better describe the phenomena reported in the paper. In particular, the analysis that were performed on the IAT data collected in T1 are included together with the analysis concerning the internal consistency of the explicit questionnaire, the analysis performed on the Explicit Δd-score, and the one performed on the Explicit data collected in T1. The last paragraph regards the correlation analysis between implicit and explicit data. (PDF) [file pone.0187043.s003.pdf]

1 Statistical Supplementary Materials

The followings paragraphs reported supplementary analysis that were performed to better frame the phenomena reported in the paper: the former regarded the IAT implicit measure, the latter the explicit measure. The conclusive paragraph reported the correlation existing between the two measures.

1.1 IAT

1.1.1 UniAnova with IAT d-scores in T1

Aimed at verifying the differences featuring the groups we studied, we analysed the IAT d-scores at T1. Such a dependent variable was analysed using a Univariate Analysis of Variance; the accounted factors were the ones described in the text (i.e., *Presence* and *Setting*). Results indicate that the Football clubs showed higher level of implicit attitude (M = .74, sd = .36) than School classes (M = .57, sd = .40; $F(1, 112) = 5.145$, $\eta_p^2 = .043$, $p < .005$). On the contrary, the analysis reported that neither *Presence* or the two by two interaction were significant ($F(1, 112) = 1.37$, $\eta_p^2 = .012$, $p = .24$; $F(1, 112) = 1.52$, *Partial* η_p^2 = .013, $p = .22$, respectively. See Fig1) .

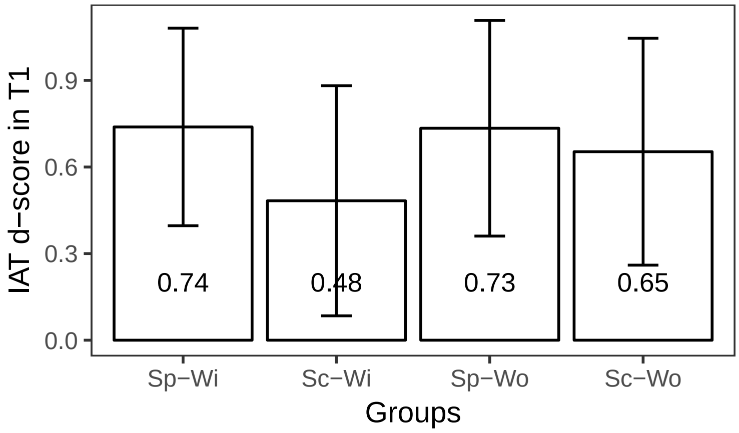

Fig. 1: The figure displays the implicit d-score expressed in T1 by each group. Standard deviation values are graphically reported. (Sp-Wi: Sport with team-mate with disability; Sc-Wi: School within classmate with disability; Sp-Wo: Sport without team-mate without disability: Sc-Wo: School without classmate with disability.)

1.2 Explicit questionnaire

1.2.1 Internal consistency analysis

The statements composing the explicit questionnaire were sorted out from a wider numbers of items tested during a pilot investigation concerning the saliency of disabilities-related concepts. Cronbach's α indexes suggested a quite low level

of internal consistency: respectively, the α index were .35 and .20 for each of the two time measures, i.e., T1 and T2, respectively.

1.2.2 UniAnova with Explicit $Delta_{d-scores}$

The Explicit $\Delta_{d-score}$ analysis reveals that no difference emerged between the settings (Football: M = .06, sd = .13; School: M = -.06, sd = .12; F(1, 112) = 1.85, η_p^2 = .02, p = *ns*), or as a function of the presence of the team/schoolmate with disability (Presence: M = .003, sd = .10; Absence: M = -.002, sd = .14; F(1, 112) = .001, η_p^2 < .001, p = *ns*), or even in the two by two interaction (F(1, 112) = .03, η_p^2 < .001, p = *ns*; See Fig2).

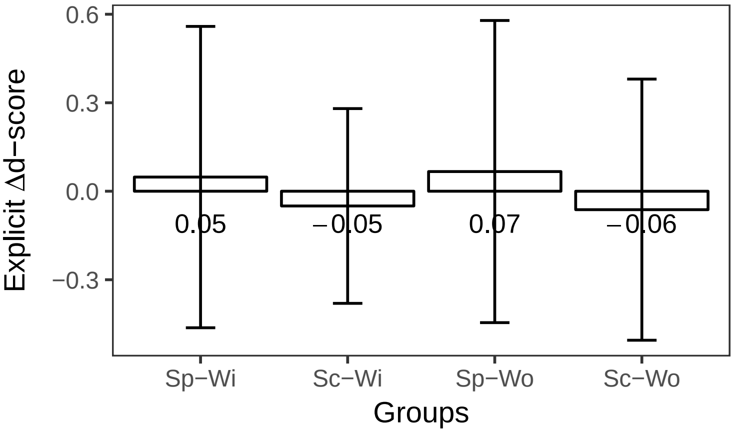

Fig. 2: The figure displays the averaged difference in the explicit attitude scores calculated between T1 and T2. Standard deviation values are graphically reported.

1.2.3 UniAnova with Explicit d-scores in T1

In order to verify the existence of a starting difference among the groups in T1, the total explicit scores expressed by each participant were analysed by using UniAnova with the same factors previously described. The results indicated the school setting revealed higher explicit attitude than the sport one (Football: M = 2.79, sd = .40; School: M = 2.98, sd = .34; F(1, 112) = 8.01, η_p^2 = .06, p < .01), no difference emerged as a function of presence/absence of the team/schoolmate among the groups (Presence: M = 2.85, sd = .38; Absence: M = 2.91, sd = .39; F(1, 112) = .84, η_p^2 = .01, p = *ns*). Moreover, by using Tukey post-hoc analysis the significant two by two interaction (F(1, 112) = 11.12, η_p^2 = .08, p < .005, See Fig3) reveals that the significant comparisons were within the school setting (Sc-Wi and Sc-Wo; p < .05) and between the two settings without team/classmate with disability (Sc-Wo and Sp-Wo: p < .001).

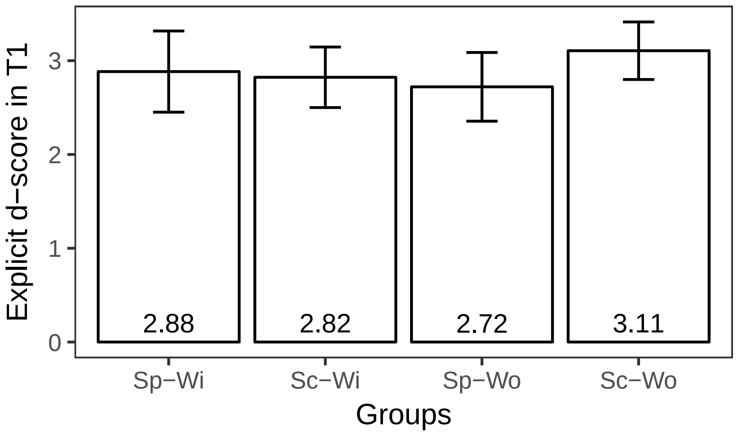

Fig. 3: The figure displays the explicit attitude score in T1 expressed in each group. Standard deviation values are graphically reported.

2 Correlation analysis between IAT and explicit questionnaire data

Finally we performed a correlation analysis to assess whether differences in the two, implicit and explicit, measurements correlated. We measured the correlation either in T1, T2 and in the respective d-scores. Finally, as one could expect, by evaluating the correlations existing between implicit d-scores and explicit ones, it emerged that the two tests' results did not correlate, either in T1, or in T2 ($Ps > .05$).
